# IgG4-related eosinophilic pleural effusion: a case report

**DOI:** 10.1186/s12877-022-03594-3

**Published:** 2023-01-19

**Authors:** Lina Wang, Jiting Di, Junfang Huang, Cuiyan Guo

**Affiliations:** 1 Geriatric Department, Peking University First Hospital, Xicheng District, Xishiku Avenue No 8, 100034 Beijing, China; 2 Department of Pathology, Peking University First Hospital, Xicheng District, Xishiku Avenue No 8, 100034 Beijing, China; 3 Department of respiratory and critical care medicine, Peking University First Hospital, Xicheng District, Xishiku Avenue No 8, 100034 Beijing, China

**Keywords:** IgG4-related disease, Eosinophilic pleural effusion, Elderly, Case report

## Abstract

**Background:**

The diagnosis of unilateral eosinophilic pleural effusion (EPE) is difficult, especially for the elderly. IgG4-related disease (IgG4-RD) is a rare cause of EPE.

**Case presentation:**

An 81-year-old man was admitted to the hospital for dyspnea due to right pleural effusion. Laboratory examination shows elevated IgG4 and eosinophils in both serum and pleural fluid. The patient was diagnosed with IgG4-RD by video-assisted thoracoscopy and pleural biopsy. We found no evidence of other organ involvement except for the EPE and history of prurigo. He was treated with prednisolone 40 mg daily orally and pleural effusion decreased significantly.

**Conclusion:**

IgG4-RD should be considered in the differential diagnosis of EPE in the elderly. High effusion IgG4 concentration may be an indication of IgG4-related pleural lesions.

## Background

Pleural effusion is common in routine clinical practice and can be due to many different diseases. The most common causes of pleural effusion are congestive heart failure, cancer, pneumonia, and pulmonary embolism 1. Eosinophils in the pleural fluid have been of interest to clinicians since the first case report in 1894 [2]. EPE is defined by an eosinophil count of ≥ 10% in the pleural fluid 3 and accounts for 5 to 16% of exudative pleural effusions 4. Common causes of EPE include malignant tumor, infection, trauma, parasite, allergy, etc. Adelman et al. 5 reviewed 343 cases of EPE in 1984 and found that the presence of pleural fluid eosinophilia considerably reduced the probability of malignancy or tuberculosis and increased the likelihood of an underlying benign disorder. Due to the comorbidity of the elderly, the differential diagnosis of pleural effusion is difficult. A few studies found that the proportion of eosinophils in IgG4 related pleural effusion seemed to increase 6. Unilateral eosinophilic pleural effusion caused by IgG4-RD is rarely reported. To our knowledge, this is the first case report of IgG4-related eosinophilic pleural effusion as the main clinical manifestation.

## Case presentation

An 81-year-old man was admitted to the hospital for dyspnea due to unexplained pleural effusion. He had a medical history of chronic urticaria and denied smoking, alcohol consumption and use of long-term drugs before. A little of right pleural effusion was found by chest CT occasionally in the routine examination six month ago. However, he experienced gradual dyspnea on exertion and the chest X-ray showed increases in the amounts of right pleural effusion one month before admission. In the development of the disease, he denied fever, chronic cough, arthralgia, abdominal pain and oral ulceration.

On examination, his oxygen saturation was 96% on room air; his heart rate was 72 beats per minute; his respiratory rate was 18 breaths per minute; his temperature was 36.5℃, and his BP was 125/76mmHg. During hospitalization, we observed that the respiratory rate accelerated to 25 breaths per minute and the SpO2 reduced to about 92% when he take a bit of light exercise. Examination of the chest revealed reduced breath sounds in the right hemithorax. Laboratory results were as follows: WBC count, 5.6 × 10^9^ cells/L; eosinophil count, 0.7 × 10^9^ cells/L (normal range: 0.02–0.52 × 10^9^ cells/L); C-reactive protein, 4.41 mg/L (normal range: 0-3 mg/L); serum IgG level 1860 mg/dl, including 848 mg/dl of IgG4; serum immunoglobulin E level 892kU/L(normal range: less than 100 kU/L); pro-gastrin-releasing peptide (ProGRP), 72.82pg/ml (normal range: less than 69.2pg/ml), other tumor markers were negative; speckled-homogenous antinuclear antibody was positive(1:1000), rheumatoid factor and anti-cyclic citrullinated peptide antibody were negative. Pleural fluid was yellow and turbid in appearance. Laboratory analysis revealed the following results: total protein, 7.30 g/dl (serum protein, 8.04 g/dl); lactate dehydrogenase (LDH),173units/L (serum LDH, 274 units/L); glucose, 9.31 mmol/L; adenosine deaminase (ADA) of 34 units/L; Pleural effusion showed a total nucleated cell count of 1702/µl (eosinophil 63%). According to the Light’s criteria, the pleural fluid was considered as exudate pleural effusion. Bacterial culture, polymerase chain reaction analysis for Mycobacterium tuberculosis and cytology of the pleural fluids were all negative. The IgG4 level of the pleural effusion was 1160 mg/dl.

**Fig. 1:**
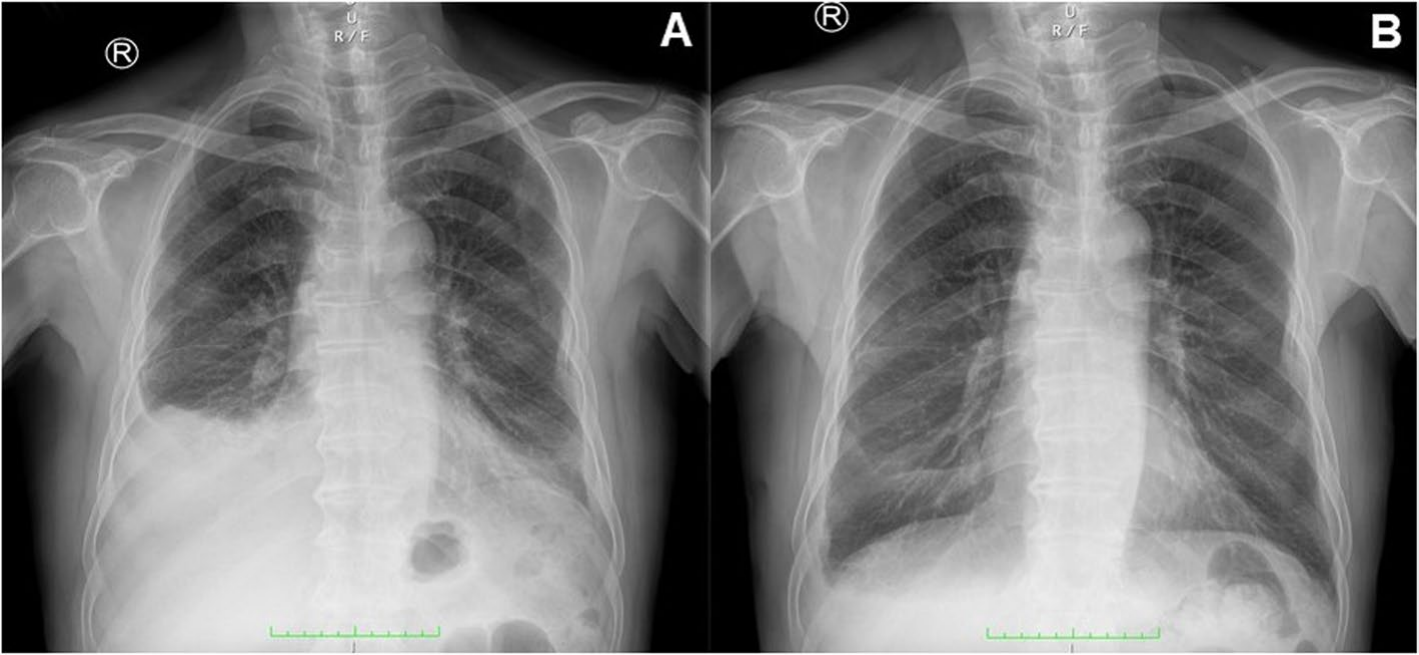
Chest radiography image of the patient

**Fig. 2:**
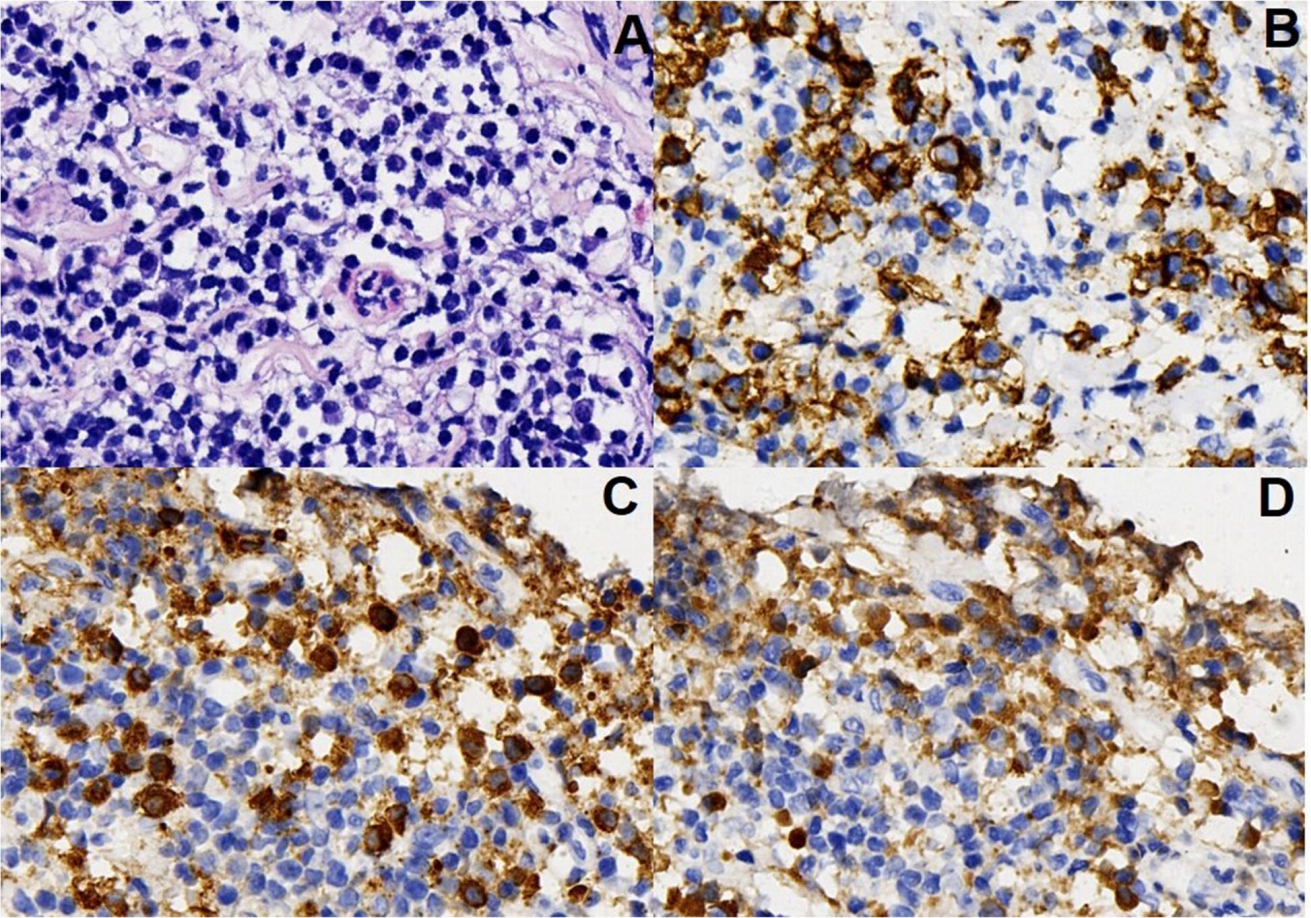
Histopathologic examination of biopsy specimens from the right pleura. **A**: Hematoxylin–Eosin staining, 40×; **B**: Immunohistochemical staining for cluster of differentiation 138, 40×; **C**: Immunohistochemical staining for IgG4(40×) showing IgG4-positive plasma cells > 50/HPF; **D**: Immunohistochemical staining for IgG (40×) showing IgG4/IgG cell ratio > 40%

Chest radiograph revealed a moderate amount of pleural effusion in the right-side chest on admission (Fig. 1 A). No abnormal findings were detected by FDG-PET/CT. We performed a thoracoscopic pleural biopsy on the third day of hospitalization. It was found that parietal pleura and diaphragmatic surface were congested, thickened and slightly adhered under thoracoscopy. Histological analysis presented a large number of lymphoid and plasma cells infiltrated and scattered eosinophils in parietal pleura. Immunohistochemical staining showed 50 IgG4-positive plasma cells per high-power field and IgG4/IgG ratio was over 40% (Fig. 2).

**Fig. 3:**
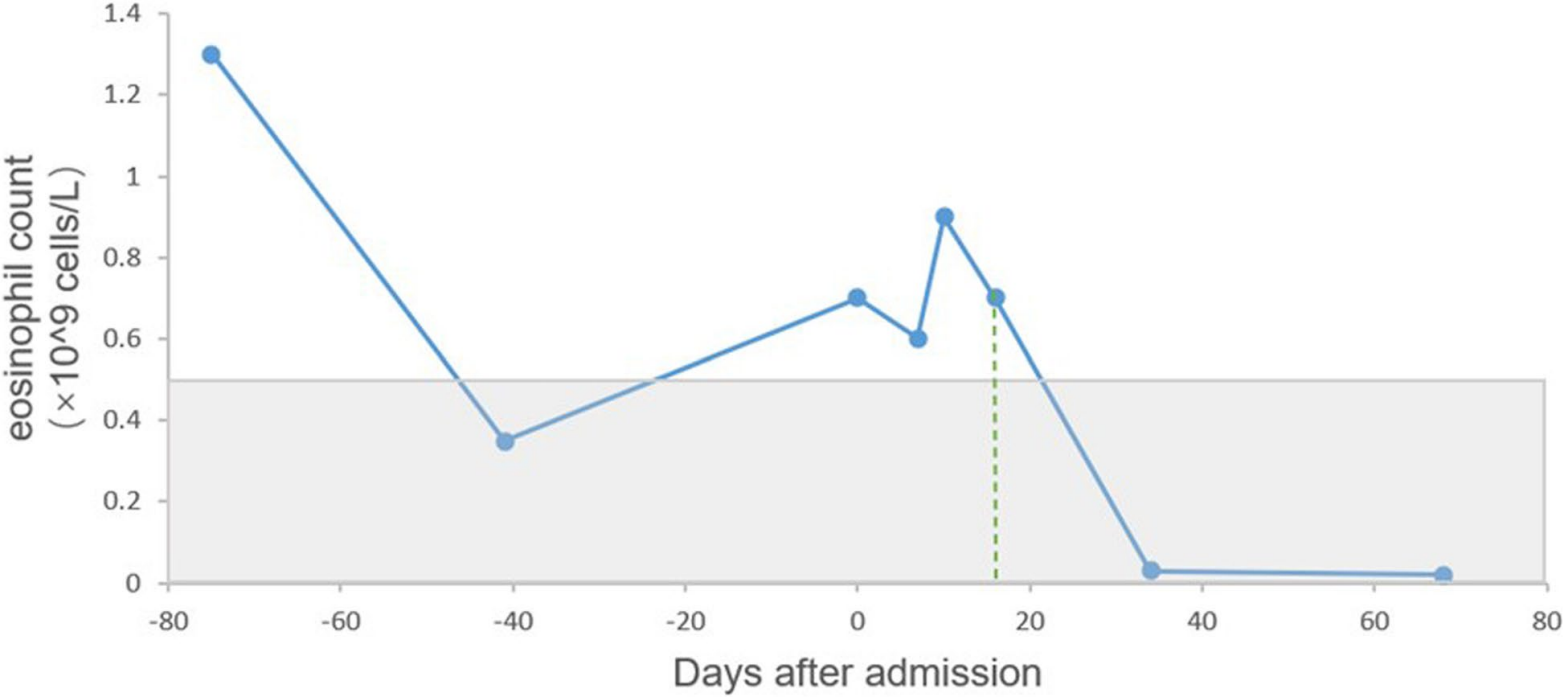
Change of the peripheral blood eosinophil count (normal range (light-gray): 0.02–0.52 × 10^9 cells/L). Day 0 refers to admission day. The patient started prednisolone on days 17 after admission(green dotted line). He was followed up as an outpatient on days 34 and 68

The patient was treated with prednisolone 40 mg daily orally initially. On review in the outpatient clinic a half month later, the pleural effusion almost disappeared in the chest radiograph (Fig. 1B) and the symptom of dyspnea was relieved. We monitored the eosinophil count and found that the eosinophil count in peripheral blood decreased significantly after the use of oral corticosteroids (Fig. 3). Prednisolone was decreased by 5 mg per week.

## Discussion and conclusion

IgG4-RD is an uncommon immune-mediated disorder that can cause fibroinflammatory lesions in nearly any organ 7. The mean age at onset was 50.3 ± 14.9 years and the majority of patients were men (60.8%) 8. Intrathoracic lesions in patients with IgG4-RD include bronchial thickening, nodules, ground glass shadow, pleural thickening/effusion, lymphadenectasis and retromediastinal fibrosis. Yunyun Fei et al. [9] found that only 4/87(4.6%) patients present pleural effusion in IgG4-RD patients with intrathoracic involvement. However, Kasashima et al. [6]has shown that pleural effusion may be the main manifestation of IgG4 related pleural lesions. They reported that 6/8 (75%) patients with IgG4-related pleural lesion had pleural effusion.

At present, the pathogenesis of IgG4-RD is not clear. Previous studies show immune-mediated mechanisms triggered by autoimmune and infectious factors may contribute to the fibroinflammatory process. Type 2 helper T cells and Tregs overexpress cytokines including interleukins (IL-4, IL-5, IL-10, and IL-13) and transforming growth factor β, promoting eosinophilia and leading to increased serum IgG4 and IgE, and progression of fibrosis 10. Eosinophilia in IgG4-RD is generally viewed as an integral part of the clinical feature. One observational study found that IgG4-pleural effusion patients have potential allergic diseases 6. This patient also had a history of chronic urticaria and present elevated serum IgE level and eosinophil counts in pleural fluid and serum. However, Zhang et al. [11] found that eosinophilia appeared independent of allergies in IgG4-RD. The causes of eosinophilia in IgG4-RD are not clear at present. Previous studies suggested that eosinophilia was probably induced by a process inherent to IgG4-RD immune response itself. There was homology between human carbonic anhydrase II and the α-carbonic anhydrase of *Helicobacter pylori*, and between the plasminogen-binding protein of *H. pylori* and the ubiquitin-protein ligase E3 component n-recognin 2 expressed in pancreatic acinar cells 12. These innate immune responses against microbial antigens may be associated with the eosinophilia in IgG4-RD.

This elderly patient showed unilateral EPE as the main manifestation, and was highly suspected of malignancy. However, the previous study found that malignant pleural effusion usually present lower (< or = 40%) pleural fluid eosinophil percentage 13. And there was no evidence of tumor by FDG-PET/CT and cytological examination of pleural effusion. Collagen-vascular disease related interstitial pneumonia is another usual cause of unilateral pleural effusion. The patient had no rash, arthralgia or other symptoms. Positive antinuclear antibody alone was not specific for the diagnosis of collagen vascular disease. Although IgG4-RD is an important cause of reactive or secondary eosinophilia, IgG4-related pleural effusion in previous cases was dominated by lymphocytes 14. However, the proportion of eosinophils predominated in pleural fluid in our patient. IgG4-RD should be considered in the differential diagnosis of EPE in future.

The diagnosis of IgG4-RD depends on the typical pathology features including the lymphoplasmacytic infiltrate, a storiform pattern of fibrosis, obliterative phlebitis and increased numbers of IgG4+ plasma cells 15. However, either storiform fibrosis or obliterative phlebitis may not be a prominent feature in IgG4-related pleural lesions 16. Clinicopathological correlation is needed when pleural effusion is the only manifestation of the disease. Elevated serum IgG4 (XX135 mg/dl) was once considered to be a diagnostic marker of the disease, but serum concentrations of this immunoglobulin are normal in up to 40% of patients with biopsy-proven IgG4-related disease 15. Some researchers noticed that pleural effusion IgG4 levels were even higher than serum IgG4 levels in 5/9 patients (56%) [14]. Murataet et al. [17] reported that 12 of 35 (34%) patients with unknown pleural effusions were found to have marked IgG4-positive plasma cell infiltration in the pleura and confirmed that effusion IgG4 levels were significantly higher in the IgG4+ group than in the IgG4^−^ group (median 54 versus 27 mg/dl, *P* < 0.01). High effusion IgG4 concentration may be an indication of IgG4-related pleural lesions.

In summary, IgG4-RD is a rare cause of unexplained eosinophilic pleural effusion. Clinicians should be alert to the possibility of IgG4-related pleural effusion in the elderly in condition of excluding other reasons carefully.

## Data Availability

All relevant data has been presented in the manuscript and further inquiry can be directed to the corresponding author.

## Abbreviations

EPE: eosinophilic pleural effusion
IgG4-RD: IgG4-related disease
ProGRP: pro-gastrin-releasing peptide
LDH: lactate dehydrogenase
ADA: adenosine deaminase
